# Neural and behavioral substrates of subtypes of Parkinson’s disease

**DOI:** 10.3389/fnsys.2013.00117

**Published:** 2013-12-24

**Authors:** Ahmed A. Moustafa, Michele Poletti

**Affiliations:** ^1^Department of Veterans Affairs, New Jersey Health Care SystemEast Orange, NJ, USA; ^2^School of Social Sciences and Psychology and Marcs Institute for Brain and Behaviour, University of Western SydneySydney, NSW, Australia; ^3^Department of Mental Health and Pathological Addiction, AUSL of Reggio EmiliaReggio Emilia, Italy

**Keywords:** Parkinson’s disease, tremor, dementia, mild cognitive impairment, hallucinations, depression, impulse control disorders

## Abstract

Parkinson’s disease (PD) is a neurological disorder, associated with rigidity, bradykinesia, and resting tremor, among other motor symptoms. In addition, patients with PD also show cognitive and psychiatric dysfunction, including dementia, mild cognitive impairment (MCI), depression, hallucinations, among others. Interestingly, the occurrence of these symptoms—motor, cognitive, and psychiatric—vary among individuals, such that a subgroup of PD patients might show some of the symptoms, but another subgroup does not. This has prompted neurologists and scientists to subtype PD patients depending on the severity of symptoms they show. Neural studies have also mapped different motor, cognitive, and psychiatric symptoms in PD to different brain networks. In this review, we discuss the neural and behavioral substrates of most common subtypes of PD patients, that are related to the occurrence of: (a) resting tremor (vs. nontremor-dominant); (b) MCI; (c) dementia; (d) impulse control disorders (ICD); (e) depression; and/or (f) hallucinations. We end by discussing the relationship among subtypes of PD subgroups, and the relationship among motor, cognitive, psychiatric factors in PD.

## Introduction

Parkinson’s disease (PD) is a neurodegenerative disorder characterized by motor (bradykinesia, rigidity and resting tremor) and non-motor symptoms, such as cognitive impairment, autonomic, affective and behavioral disturbances (Jankovic, 2008). The complex clinical picture of motor and non-motor symptoms is not only different between PD patients, but is also changing along the course of disease progression in each patient. The neurodegenerative nature of PD and its pharmacological management are involved in this clinical heterogeneity. PD is characterized by a progressive widespread diffusion of the Lewy body neuropathology from subcortical to cortical structures (Braak et al., 2003); therefore at different disease stages PD patients present different loads of Lewy body neuropathology and different involvements of subcortical and cortical structures. Moreover, drugs used to manage clinical symptoms, as dopaminergic drugs (Bonuccelli and Pavese, 2006; Poewe et al., 2010) have motor and non-motor effects that change along disease progression (Poletti and Bonuccelli, 2013).

Classifications of PD into different subtypes have been proposed to reduce the heterogeneity of clinical features associated with PD, and thus better investigate their neural correlates and provide better treatment options. Two approaches are used to achieve these classifications: empirically assigned or data-driven (Marras and Lang, 2013). Empirically assigned classifications of specific clinical motor and non-motor symptoms in PD patients (e.g., rigidity, cognitive impairment, psychosis, impulse control disorder (ICD), autonomic dysfunction) compare samples of patients with vs. without the investigated clinical symptom; for example, on the basis of the predominant motor symptoms as indicated by the Unified Parkinson’s Disease Rating Scale (UPDRS) motor subscores, PD patients were categorized into patients with predominant postural instability and gait difficulty and patients with predominant tremor (Jankovic et al., 1990). On the other hand, the data-driven approach searches for variables that group together each subtype without a-priori hypothesis; for example the cluster analysis of Lewis et al. (2005) identified four subtypes of PD patients: (1) young onset; (2) non-tremor dominant with cognitive impairment and depression; (3) rapid progression without cognitive impairment; and (4) tremor dominant.

The empirically assigned and the data-driven classification approaches may identify partially overlapping subtypes (van Rooden et al., 2010), as in the case of the motor subtypes proposed by Jankovic et al. (1990) and clusters proposed by Lewis et al. (2005). These approaches have been attempted for motor, cognitive and psychopathological features of PD patients, while, they have not been attempted on findings of neuropathological and neuroimaging assessments.

A recent comparison of the empirically assigned and data driven classification approaches (Marras and Lang, 2013) underlined how the former has the advantages of small number of subtypes, ease of implementation and assignment of patients to one or another subtype; on the contrary clusters derived from the data-driven approach are inherently more complicated, incorporating more variables that are often not regularly measured in clinical practice, increasing the difficulty to assign some patients to subtypes.

Furthermore the empirically assigned classification is probably more informative on the pathophysiology of specific PD symptoms but it could also hamper a global view on complex clinical patterns of motor and non-motor symptoms that characterize PD patients. Since data driven subtypes reviewed elsewhere (van Rooden et al., 2010), this review aims at presenting an overview of some of the main PD subtypes derived from the empirically assigned classification; we briefly present their neural and behavioral correlates to show how these subtypes may correlate with each other. For this purpose in the next sections we describe the following subtypes: motor (postural instability and gait difficulty vs. tremor) and non-motor symptoms (mild cognitive impairment (MCI) vs. dementia; with/without ICD, depression, or hallucinations). Next, we briefly discuss existing data on how these subtypes correlate with each other.

## Cognitive subtypes in PD: from mild cognitive impairment to dementia

Since the early clinical stages, PD patients present an increased risk of cognitive impairment, with prevalences of MCI ranging from 14.8–18.9% in newly diagnosed drug-naïve patients (Aarsland et al., 2009, 2010; Poletti et al., 2012a,b), up to a 50% and 5 years after clinical diagnosis (Broeders et al., 2013).

MCI may be diagnosed when a neuropsychological impairment is demonstrated by performances 1–2 standard deviations below appropriate norms in at least two tests of the same cognitive domain (MCI single domain; for example dysexecutive MCI when only the executive domain is impaired; amnestic MCI when only episodic memory is impaired) or in at least one test in two different cognitive domains (MCI multiple domains: for example executive and episodic memory domains or executive and visuospatial domains are impaired) and there is a preserved functional level in everyday activities, not considering difficulties related to the motor symptoms (Litvan et al., 2012).

In the early clinical stage, cognitive impairment in PD is primarily characterized by deficits if executive functions, caused by loss of dopaminergic neurons in the nigrostriatal pathway (Kish et al., 1988) and resulting in the reduction of dopamine levels in the striatum. This dopamine reduction negatively impacts the functioning of the dorsolateral frontostriatal loop (linking dorsolateral prefrontal cortex, dorsolateral caudate nucleus of the striatum, dorsomedial globus pallidus and thalamus), which is mainly involved in of executive functions such as working memory, planning and set-shifting (Alexander et al., 1986) and dopaminergic drugs have a beneficial effects on these functions in the early stages of disease by impacting striatal dopamine levels (Cools, 2006).

The presence of MCI since the early PD stages is associated not only with the frontostriatal deficit but also with an early involvement of parietal and occipital cortices (Pappata et al., 2011); this finding have been consistently reported in patients with MCI by structural neuroimaging studies, detecting atrophic changes in a number of cortical regions, including occipital, parietal, medial temporal and prefrontal cortices (Song et al., 2011; Weintraub et al., 2011a; Lee et al., 2012a) and cortical hypometabolism in frontostriatal loops and parietal and occipital regions (Nobili et al., 2011; Ekman et al., 2012; Garcia-Garcia et al., 2012; Nagano-Saito et al., 2013).

Advanced PD stages, usually presenting dementia in 75–80% of patients (Aarsland et al., 2003; Williams-Gray et al., 2013), are characterized by widespread cortical and subcortical atrophic changes (Burton et al., 2004; Nagano-Saito et al., 2005; Summerfield et al., 2005; Beyer et al., 2007; Weintraub et al., 2011b; Melzer et al., 2012) and more severe temporal, parietal and occipital hypometabolism in comparison with MCI patients (Garcia-Garcia et al., 2012).

In sum, the cognitive profile of PD is usually characterized by an early executive impairment, a sign of nigrostriatal degeneration, and subsequently by impairment of visuospatial functions, memory and/or language, a sign of cortical diffusion of Lewy body pathology, evidenced by cortical atrophy and hypometabolismml: this second feature in particular, in comparison with the first one, is associated with an increased risk of developing MCI and subsequently dementia (Jellinger, 2013; Kehagia et al., 2013).

## Tremor vs. non-tremor in PD

As discussed above, PD involves a spectrum of motor symptoms that include akinesia, bradykinesia, and resting tremor, among others. Few studies categorize the patients into different subgroups, depending on the motor symptoms they present. This usually involves dividing the patients into a tremor-dominant and non-tremor-dominant groups, with the latter is either patients with predominant akinesia, bradykinesia, or postural instability and gait symptoms (Jankovic et al., 1990; Zaidel et al., 2009; Mure et al., 2011; Schillaci et al., 2011; Lee et al., 2012b; Wylie et al., 2012). Bradykinesia, and postural instability and gait dysfunction are more common in patients with a rapid disease progression compared with PD patients with a slower progression rate (Jankovic et al., 1990).

Studies have generally shown that PD patients with tremor are usually less cognitively impaired than PD patients with akinesia or gait dysfunction (Burn et al., 2006; Lyros et al., 2008; Oh et al., 2009; Domellof et al., 2011). For example, Vakil and Herishanu-Naaman (1998) found that tremor-dominant patients are less impaired at procedural learning tasks than akinesia-dominant patients. Studies also showed that PD patients with tremor are less impaired than PD patients with other motor subtypes on perceptual tasks, including peripheral vision and visual processing speed (Seichepine et al., 2011). Interestingly, we also found that akinesia-dominant patients were more impaired than tremor-dominant patients at various working memory (Moustafa et al., 2013a) and learning (Moustafa et al., 2013b) measures. Prior studies have reported significant correlations between bradykinesia severity and cognitive measures in newly diagnosed PD patients (Domellof et al., 2011; Poletti et al., 2012b). For example, Domellof et al. (2011) found that bradykinesia scores correlate with Wisconsin Card Sorting Test, digit span, and Trail Making Test performance. Along the same lines, (Poletti et al., 2012a,b) reported a correlation between bradykinesia and Trail Making Test as well as achieved category in the Modified Card Sorting Test.

This pattern of results also applies to psychiatric symptoms. One neuropsychological study found that unlike tremor-dominant patients, PD patients with non-tremor symptoms show increased rates of depression, apathy, and hallucinations (Reijnders et al., 2009). Further, neuropsychological studies found depression is more common in akinesia-dominant patients than tremor patients (Starkstein et al., 1998). Recently, a neuropsychological study found that unlike patients with dominant tremor symptoms, patients with postural instability and gait deficits show more impulsive behavior (Wylie et al., 2012). Clinical and neuropsychological studies also suggest that the severity of akinesia symptoms is a risk factor for the development of dementia and MCI in PD patients (Poletti et al., 2011; Poletti and Bonuccelli, 2013).

For more than two decades, it has been shown that patient with dominant akinesia show more neural damage than PD patients with dominant tremor (Paulus and Jellinger, 1991). Recent neuropsychological studies showed that non-tremor symptoms in PD, including postural instability and gait deficits, are associated with grey matter degeneration (Rosenberg-Katz et al., 2013). Other neuropsychological and animal studies suggest that akinesia and bradykinesia are arguably associated with basal ganglia (and corticostriatal circuits) dysfunction, while tremor is perhaps associated with cerebellar, thalamic, and subthalamic nucleus abnormalities (Kassubek et al., 2002; Probst-Cousin et al., 2003; Weinberger et al., 2009; Zaidel et al., 2009; Mure et al., 2011). For example, Schillaci et al. (2011) found that PD patients with akinesia and rigidity as the predominant symptoms have significantly more widespread dopamine loss in the striatum than PD patients with tremor as the predominant symptom(also see Eggers et al., 2011). These results support a relationship between motor variables (including akinesia and bradykinesia) and cognitive performance in PD patients.

## Impulse control disorders in PD

Dopaminergic medications, especially some dopamine agonists, can trigger ICDs, such as hypersexuality, hobbyism, dopamine dysregulation syndrome, binge eating and pathological gambling, in a considerable subpopulation of PD patients (Dodd et al., 2005; Voon et al., 2007). It is also important to note that ICDs can be caused by other factors beside the administration of dopaminergic medications. For example, few studies reported the occurrence of ICDs in drug-naïve PD patients (Antonini et al., 2011). Interestingly, studies also report that some PD patients present either with single or multiple ICDs, and that each of these subgroups have a different cognitive and neural profile (Vitale et al., 2011). Further, few studies have investigated the prevalence and predictors of ICDs. For example, it was reported that alexithymia is a predictor of ICDs in PD patients (Goerlich-Dobre et al., 2013). It was also found that frontal executive function is a predictor of the occurrence of pathological gambling (Santangelo et al., 2009b).

Prior studies show that ICDs are observed more often in patients on D2 dopamine agonists (Weintraub et al., 2006; Voon and Fox, 2007) It is suggested that patients vulnerable to ICDs have a lower D2 receptor density, even before onset of PD (Dagher and Robbins, 2009). The density of D2 receptors might further decreases in these vulnerable patients by overstimulation of ventral striatal D2 receptors in PD and increases the rate of ICDs in such vulnerable patients. Other studies suggest that binding to dopamine D3 receptors is responsible for the occurrence of ICDs (Vilas et al., 2012). A recent study found that patients with ICDs were more likely to be on antidepressant medications and had more motor complications than those without ICDs (Mack et al., 2013). In addition to dopaminergic medications, studies also show that ICDs can be caused by deep brain stimulation of the subthalamic nucleus (Frank et al., 2007; Callesen et al., 2013a; but also see Santangelo et al., 2013). Studies also show that cognitive behavioral therapy can ameliorate ICDs in PD patients (Okai et al., 2013).

ICDs in PD is also associated with cognitive and psychiatric symptoms. Unlike patients without ICDs, PD with ICDs show increased discounting in delay discounting tasks (Housden et al., 2010; Voon et al., 2010b; Leroi et al., 2013), increased reward learning (Voon et al., 2011), and impairment performing the Iowa gambling task (Gescheidt et al., 2012). Studies have also shown that PD patients with ICDs are more impaired than patients without ICDs on working memory (Djamshidian et al., 2010; Voon et al., 2010a), but they did not differ on executive functioning (Siri et al., 2010). A recent study also showed that PD patients with ICDs are more impaired at planning and set-shifting tasks than patients without ICDs (Vitale et al., 2011), although the study did not include a healthy control group. Another study showed ICDs in PD are associated with executive dysfunction (Voon et al., 2010b) and working memory impairment (Voon et al., 2011). Further, it was found that PD patients with the hypersexuality subtype show more impairment on the Stroop task than patients with pathological gambling (Vitale et al., 2011), suggesting that the different impulsive behaviors are associated with different behavioral, and potential neural, profile. Studies also found that ICDs in PD are associated with depression and irritability (Pontone et al., 2006).

Neural studies have implicated cortical and subcortical structure for the occurrence of ICDs in PD. Many studies show that the underlying neural substrates of ICDs in PD are mostly the ventral striatum, including the nucleus accumbens (Cools et al., 2007; Dagher and Robbins, 2009; Steeves et al., 2009; Voon et al., 2010a). For example, using PET imaging, Steeves et al. (2009) found greater decreases in binding potential in the ventral striatum in PD patients on dopamine agonists with pathological gambling. Additionally, (Voon et al., 2010a,b) reported impaired dopamine signaling in ventral striatal blood oxygen level dependent (BOLD) in PD patients with ICDs. It has been suggested that restoration of dopamine transmission in the dorsal striatum might lead to overdosing of the ventral striatum which results in excessive dopamine receptor stimulation in the ventral striatum (Swainson et al., 2000; Cools et al., 2001) that induce ICDs in some subjects (Cools et al., 2003; Dagher and Robbins, 2009). A recent study showed that increase of striatal dopamine might lead to ICDs in PD (Voon et al., 2013). Studies have also implicated the hippocampus in the occurrence of ICDs in PD (Calabresi et al., 2013). Beside subcortical structures, neural studies have shown that patients with ICDs show dopamine reduction in the ventromedial prefrontal cortex (and nucleus accumbens) compared with those without ICDs (Lee et al., 2013).

## Depression in PD

Depression is one of the most common non-motor symptoms in PD patients (Schrag et al., 2007; Picillo et al., 2009). It is estimated that roughly 40% of PD patients show depressive symptoms (Slaughter et al., 2001; McDonald et al., 2003; Leentjens, 2004; Schrag et al., 2007; clinical manifestations, etiology, and treatment of depression in PD). These depressive symptoms include social withdrawal and anhedonia (inability to experience pleasure). Depression in PD is often diagnozed as follows: patients with Beck Depression Inventory (BDI) score greater than 13/14 is considered in the depression group, while the others are in the non-depressed group (Leentjens et al., 2000; Schrag et al., 2007; Herzallah et al., 2010). Furthermore, patients with major depressive disorder have a threefold higher risk to develop Parkinson’s later in life (Schuurman et al., 2002). Like ICDs, it is well recognized that the incidence of depression among PD patients is much higher than among age-matched healthy participants (Cummings, 1992; Veiga et al., 2009). Selective serotonin reuptake inhibitors as well dopamine agonists, such as pramipexole and pergolide were shown to have antidepressant effects in PD depression (Picillo et al., 2009).

Research has shown that depressive symptoms in PD have a negative impact on PD patients’ quality of life (Karlsen et al., 1999; Schrag et al., 2000). Studies also suggest depression in PD is associated with intellectual impairment and inattention (Mayeux et al., 1981). Furthermore, depression in PD is associated with cognitive impairment (Kuzis et al., 1997; Bader and Hell, 1998; Errea and Ara, 1999). Current studies in PD patients with depression largely focus on executive functioning, working memory, or memory (Kuzis et al., 1997; Norman et al., 2002; Kummer et al., 2009; Santangelo et al., 2009a). We also found that PD patients with depression were more impaired at learning tasks than PD patients without depression (Herzallah et al., 2010).

It debated in the literature which mechanism contributes the occurrence of depression in PD, and whether depression symptoms are related to other psychiatric symptoms in PD, including apathy and anxiety. Further, it is debated whether depression in PD are related to dopamingeric dysfunction (Eskow Jaunarajs et al., 2011). Some also argue that depressive symptoms in PD can be due to psychosocial factors or secondary to motor impairment (Aarsland et al., 2012). Further, it is not clear which neurotransmitter system dysfunction contributes to PD depression, as many argue it could be related to dopaminergic, noradrenergic and/or serotonergic dysfunction (Aarsland et al., 2012). It is also argued that depressive symptoms are more related to severity of motor symptoms in PD, particularly akinesia and bradykinesia (Reijnders et al., 2009).

Neurobiological studies have also investigated the neural substrates of depression in PD. For example, imaging studies found that patients with PD who develop depression show structural changes that reflect dopaminergic dysfunction (Walter et al., 2010). This is in agreement with case reports showing that deep brain stimulation of the substrania nigra can trigger depressive symptoms in PD (Bejjani et al., 1999). Other neural studies suggest that while the primary neural dysfunction of PD is the dorsal striatum and its dopaminergic afferents (Kish et al., 1988), depression in PD is associated with deficits in the ventral regions within striatum, including the nucleus accumbens (Remy et al., 2005). Interestingly, Voon et al. (2011) argue that depression in PD (hypoactive state) is perhaps the antithesis of ICDs (hyperactive state). For example, research has shown that depression in PD can result from reduction of dopamine levels in the brain (Thobois et al., 2010). This is contrasted from ICDs, which is often associated with increased dopamine levels, due mostly to the administration of dopamine agonist therapy (Voon et al., 2007), but also see Callesen et al. (2013b) for evidence of association of impulsivity and depression in PD.

It is debated whether depression is caused by motor abnormalities or other neuropathology in PD. Imaging studies suggest that patients with depression who show structural abnormalities at the level of the substantia nigra are possibly at an elevated risk of later developing definite PD (Hoeppner et al., 2009; Shen et al., 2013). Nonmotor manifestations of PD (such as depression) are the earliest to appear (Simuni and Sethi, 2008).

## Hallucinations in PD

Psychosis and visual and olfactory hallucinations occurs in approximately 20–30% of PD patients (Rabey, 2009). In PD patients, visual hallucinations are more common than auditory or olfactory hallucinations (Diederich et al., 2009). Psychosis is usually defined as involving one of the following symptoms: (a) illusions (misinterpretations of existing stimuli), hallucinations (defined as hallucinatory symptoms), and/or delusional symptoms. Psychosis in PD is usually confirmed using the Parkinson’s Psychosis Rating Scale (Friedberg et al., 1998). Although it was previously found that the administration of dopaminergic drugs is the main cause of psychosis and hallucinations (Morgante et al., 2012), recent studies additionally show that sleep disturbance, dementia, longer disease duration, and advanced stage of the disease, can also exacerbate psychotic symptoms in PD patients (Poewe, 2003; Fenelon et al., 2006; Fenelon, 2008; Bannier et al., 2012; Gibson et al., 2013; Lee and Weintraub, 2012; Morgante et al., 2012). So unlike earlier clinical practice, a diagnosis of psychosis in PD is made when the patients have not had any dopaminergic medications. Among risk factors, one longitudinal study found that frontal dysfunction was also a predictor for the development of hallucinations in PD (Santangelo et al., 2007).

Prior reports also suggest that hallucinations in PD patients are the main reason for admission to nursing homes (Diederich et al., 2009). Psychosis in PD is also a risk factor for the occurrence of severe cognitive dysfunctions such as dementia (Factor et al., 2003) and is associated with a diminished quality of life (Zahodne and Fernandez, 2008; Rabey, 2009). Prior studies have shown strong links between medication dosage, dementia, sleep disturbance, and psychosis in PD patients (Poewe, 2003; Fenelon, 2008; Bannier et al., 2012; Gibson et al., 2013).

There have been very few studies investigating the perceptual and cognitive correlates of psychosis in PD. Studies generally report cognitive and behavioral dysfunction in PD patients with psychosis than in patients without psychosis (Baydas et al., 2005; Shine et al., 2011). For example, studies have shown that PD patients with hallucinations are more impaired than PD patients without hallucination on recognition memory (Barnes et al., 2003), executive function (Grossi et al., 2005), frontal function (as measured using the frontal assessment battery), attentional processes (Meppelink et al., 2008), and semantic fluency (Ramirez-Ruiz et al., 2006). Specifically, Botha and Carr (2012) argue that the gating of irrelevant information to working memory is the mechanism underlying the occurrence of hallucinations. Prior studies have shown that hallucinations and psychosis in PD patients are associated with visual disturbance (Gallagher and Schrag, 2012; Shine et al., 2012). Behavioral differences between PD patients with and without psychosis can be observed during the performance of complex perceptual tasks (Shine et al., 2011).

Prior studies suggest that hallucinations in PD patients can be due to either cortical or subcortical atrophy (Papapetropoulos et al., 2006). Specifically, psychosis and hallucinations can stem from dysfunction to either the prefrontal cortex (Corlett et al., 2007; Fletcher and Frith, 2008), basal ganglia (Frank, 2008; Howes et al., 2012), or hippocampal region (Bogerts et al., 1985; Weinberger, 1999; Goldman and Mitchell, 2004; Keri, 2008; Grace, 2010). Studies also report temporal lobe dysfunction in PD patients with visual hallucination (Botha and Carr, 2012) and also with studies showing that early onset of hallucinations in PD patients is associated with dysfunction to the parahippocampus and inferior temporal cortex (Harding et al., 2002). It was found that carrying the* APOE* ∈4 allele, which is associated with a small hippocampal volume in healthy older subjects (Alexopoulos et al., 2011), is also a risk factor for the development of psychotic episodes in PD patients (de la Fuente-Fernandez et al., 1999; Goldman et al., 2004; Feldman et al., 2006).

## Discussion: relationships among PD subtypes

In the previous sections we briefly reviewed some clinical subtypes of PD derived from the empirically assigned classification approach, that has the advantage of describing a small number of subtypes as regards specific clinical symptoms, with ease of implementation and assignment of patients to one or another subtype. On the other hand, because of inability to investigate relationships between subtypes, this approach does not provide a global view on complex clinical patterns of motor and non-motor symptoms.

This study aimed at reviewing most common PD subtypes derived from the empirically assigned classification to attempt a possible integration of them, through the identification of their possible relationships and their possible common neuropathological causes Indeed the clinical heterogeneity of PD lead to the classification in many subtypes as regards motor symptoms (e.g., postural instability and gait difficulty vs. tremor), cognition (MCI vs. dementia), psychopathological features (e.g., with ICD vs. without ICD; with psychosis vs. without psychosis), demographic features (e.g., young onset vs. late onset) and disease features (e.g., rapid progression vs. slow progression).

A first issue involves the relationship between motor subtypes and subtypes of non-motor symptoms, in particular of the cognitive and psychopathological domains. The non-tremor dominant motor subtype usually presents a more severe clinical pattern of non-motor symptoms in comparison to the tremor dominant subtype. Since the early untreated stages of PD, this subtype is characterized by a higher risk of MCI (Poletti et al., 2012a) and longitudinally is associated with faster motor worsening (Vu et al., 2012), cognitive decline (Burn et al., 2006), and higher risk of developing dementia (Alves et al., 2006; Sollinger et al., 2010). This subtype is also associated with an increasing risk of developing psychopathological features, including affective features such as depression and alexithymia (Starkstein et al., 1998; Reijnders et al., 2009; Poletti et al., 2011; Burn et al., 2012) and psychotic features as hallucinations (Reijnders et al., 2009). Conversely, the tremor dominant subtype appears to be characterized by a less severe clinical picture, with a slower progression of motor and cognitive symptoms and a lower risk of developing dementia and psychopathological features. The more severe clinical picture of the non-tremor dominant PD subtypes is due to a more severe Lewy body neuropathological load, as found at the post-mortem pathological examination (Selikhova et al., 2009) and a more severe gray matter atrophy of cortical and limbic structures, as indicated by neuroimaging studies (Rosenberg-Katz et al., 2013).

A second issue is regarding the relationship between cognitive subtypes and psychopathological subtypes. Few empirical findings are available from studies based on the classification of cognitive subtypes: only two studies directly compared cognitive subtypes of PD patients in relation to other non-motor subtypes. One study compared 54 cognitively preserved patients, 48 PD patients with MCI and 25 PD patients with dementia (Leroi et al., 2012). Apathy was reported in almost 50% of MCI patients and PD patients with dementia, and was the only psychopathological manifestation differentiating cognitively preserved patients from MCI patients. Moreover the prevalence of psychotic symptoms as hallucinations and delusion progressively increased according to the degree of cognitive impairment (12.9% in cognitively preserved, 16.7% in MCI and 48% in PD patients with dementia). Another study compared different subtypes of MCI (Goldman et al., 2012) in 128 PD patients with MCI; according to the cognitive profile patients were classified as non-amnestic single domain (47.7% of the sample), amnestic multiple domain (24.2%), amnestic single domain (18.8%), and non-amnestic single domain, and executive functions and visuospatial functions were the most frequently impaired domains. In comparison to other subtypes, non-amnestic multiple domain MCI patients showed most pronounced difficulties with postural instability and gait; subtypes did not differ in relation to age, PD duration, medication use, mood or behavioral disturbances.

These few findings are in agreement with the more robust empirical literature on the classification in psychopathological subtypes, suggesting that different subtypes may present different clinical patterns along the disease course. The subtype characterized by affective features (e.g., depression, apathy and anxiety) is very common at each disease stage, because it is associated with several non-disease specific risk factors (Leentjens et al., 2013); moreover it is also associated with the executive impairment (Poletti et al., 2012a), and it is only partially modified by dopaminergic therapies (Eskow Jaunarajs et al., 2011).

Different patterns characterize psychotic features as hallucinations and delusions. Hallucinations may be present in patients with MCI (Shin et al., 2012; Hepp et al., 2013) but are particularly prevalent in advanced PD stages, in association with dementia (Rana et al., 2012) and with cortical atrophy at neuroimaging (Papapetropoulos et al., 2006). Delusions also may be associated with hallucinations and dementia in advanced patients but may be present in isolation in cognitively preserved patients, probably due to the interaction between individual susceptibility (as psychiatric familial history) and dopaminergic therapy (Poletti et al., 2012a).

ICD are more in common in patients without dementia than in patients with dementia (9.6% vs. 3.8% in a recent cross-sectional study: Poletti et al., 2013). However, an impairment of both orbitofrontal and dorsolateral executive functions may represent a risk factor (Poletti and Bonuccelli, 2012) and probably interplays with individual (premorbid level of impulsivity), pharmacological (dopamine agonist therapy) and disease related (striatal dopaminergic alteration) risk factors (Dagher and Robbins, 2009).

A controversial issue involving motor, cognitive and psychopathological PD subtypes regards the possible influence of the side of motor onset. Empirical findings principally involve cognition: the side of motor onset does not influence cognition in newly diagnosed untreated patients (Erro et al., 2013; Poletti et al., 2013); in patients “on” dopaminergic therapy a right-side motor symptom predominance is typically associated with difficulties in tasks of language and verbal memory, whereas a left-side motor symptom predominance is typically associated with difficulties in visuospatial tasks (Verreyt et al., 2011). More heterogeneous and controversial are findings on the role of the side of motor onset on motor subtypes (e.g., Stewart et al., 2009; Baumann et al., 2013) and especially on psychopathological subtypes (e.g., Foster et al., 2011; Dewey et al., 2012), therefore more empirical studies are needed on this issue.

Further empirical studies are also needed on other clinical features that have been scarcely investigated in relation to motor, cognitive and psychopathological subtypes. For example, data driven classification methods (van Rooden et al., 2010) suggested that PD patients could be also classified as “rapid disease progression” vs. “slow disease progression” or according to age of PD onset (e.g., young vs. late onset); in this perspective these features could be investigated by studies based on empirically assigned classifications. Moreover also other features suggested by empirically assigned classifications, as freezing of gait and autonomic dysfunctions, should be investigated in relation to motor, cognitive and psychopathological subtypes.

In sum, all these psychopathological subtypes may be present in all disease stages, and the presence of cognitive impairment represents a risk factor for their occurrence. Overall, PD is a heterogeneous disorder encompassing many subtypes along motor, cognitive, and psychiatric dimensions. Further, as discussed here, some of these subtypes are related (e.g., isolated delusions and ICD are more common in patients without dementia while hallucinations are more common in patients with dementia).

## Conflict of interest statement

The authors declare that the research was conducted in the absence of any commercial or financial relationships that could be construed as a potential conflict of interest.
